# Mediastinal paraganglioma successfully resected by robot-assisted thoracoscopic surgery with en bloc chest wall resection: a case report

**DOI:** 10.1186/s12893-020-00701-2

**Published:** 2020-03-05

**Authors:** Hiroaki Shidei, Hideyuki Maeda, Tamami Isaka, Takako Matsumoto, Tomoko Yamamoto, Yoji Nagashima, Masato Kanzaki

**Affiliations:** 1 Department of Thoracic Surgery, Tokyo Women’s Medical University, 8-1 Kawada-cho, Shinjuku-ku, Tokyo, 162-8666 Japan; 2 Department of Surgical Pathology, Tokyo Women’s Medical University, 8-1 Kawada-cho, Shinjuku-ku, Tokyo, 162-8666 Japan

**Keywords:** Mediastinal tumor, Robot-assisted thoracoscopic surgery, Paraganglioma

## Abstract

**Background:**

Robot-assisted thoracoscopic surgery (RATS) is useful for surgery in the apical region of the chest cavity, as it narrows towards the head. Here, we describe a nonfunctional, rib-invasive paraganglioma arising in the posterior mediastinum that was successfully removed using RATS combined with chest wall resection.

**Case presentation:**

A 31-year-old woman presented with a posterior mediastinal mass on chest computed tomography (CT) scan during a medical check-up 2 years prior. Positron emission tomography/computed tomography scan with F-18 fluorodeoxyglucose revealed a mass associated with standardized uptake maximum value of 2.69. With a preoperative diagnosis of neurogenic tumor by CT-guided percutaneous fine-needle aspiration biopsy, we performed robot-assisted tumor resection combined with chest wall resection. The wristed instruments of the robotic surgical system have increased range of motion and enabled the tumor resection without organ injury in the thoracic cavity. Histopathology examination revealed a non-functional paraganglioma with rib invasion.

**Conclusions:**

RATS is a useful technique, enabling safer and easier resection of a mediastinal tumor adjacent to surrounding organs.

## Background

Mediastinal paragangliomas are rare tumors that arise from extra-adrenal paraganglionic cells of the sympathetic nerve system; they are commonly known as pheochromocytomas. Paragangliomas are classified as functional or nonfunctional based on their ability to synthesize and release catecholamines.

Robot-assisted thoracoscopic surgery (RATS) has been used to overcome the limitations of video-assisted thoracoscopic surgery with favorable results for mediastinal tumors; the number of RATS procedures has increased rapidly in Japan [1, 2]. Here, we report the case of a nonfunctional, rib-invasive paraganglioma arising in the posterior mediastinum that we removed using RATS combined with chest wall resection. To the best of our knowledge, this is the first reported use of RATS for a posterior mediastinal tumor combined with chest wall resection.

## Case presentation

**Fig. 1:**
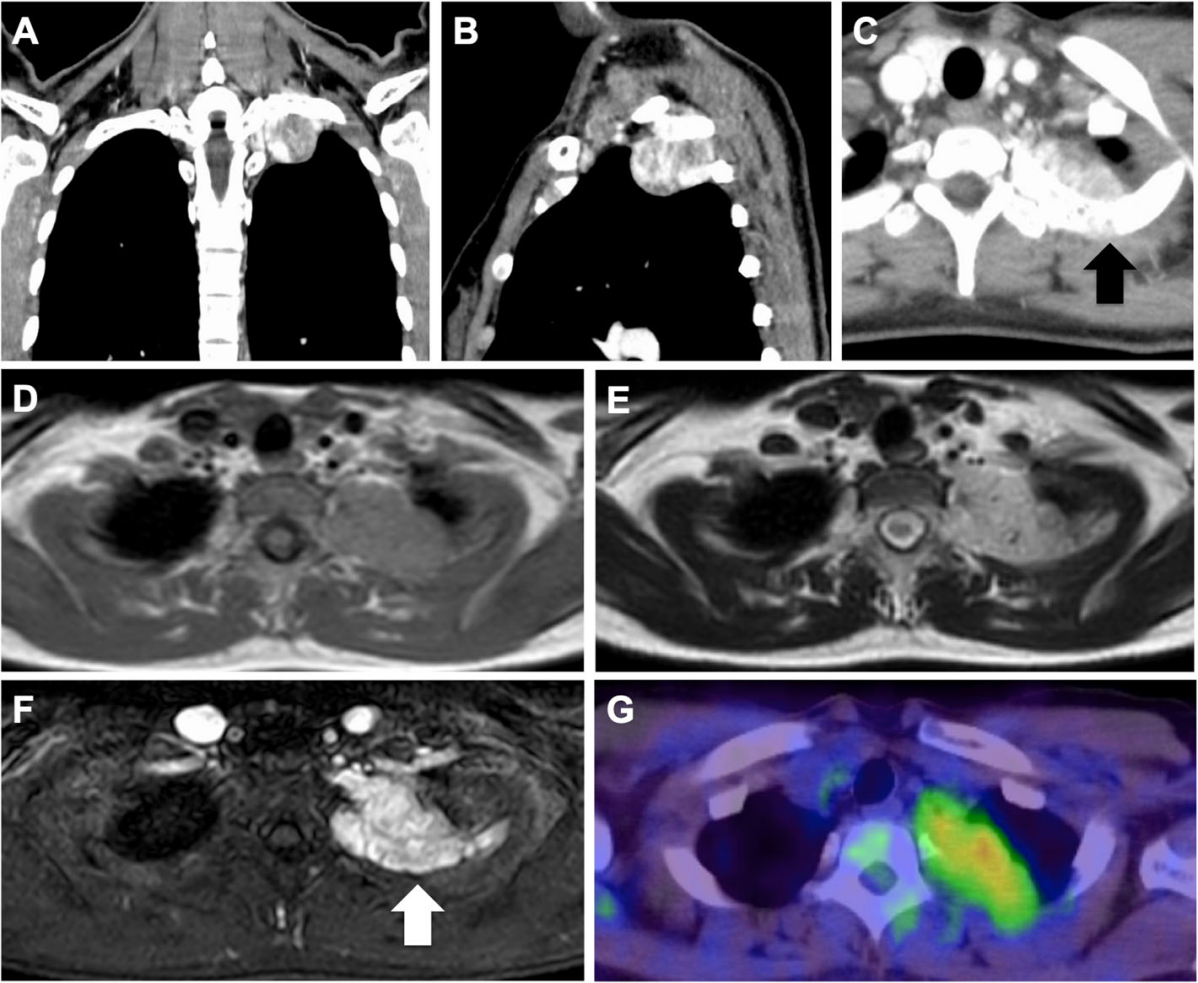
**a** Contrast-enhanced computed tomography revealing a solitary nonhomogeneous 4-cm mass in the posterior mediastinum. **b, c** The mass invaded the left 2nd rib (black arrow). **d, e** Magnetic resonance imaging (MRI) showing that the signal inside the mass was representative of muscle tissues equivalent to that on the T1-weighted image and hyperintense on the T2-weighted image. **f** Contrast-enhanced MRI showing similar signal changes and contrast effects on the dorsal side of the second rib in contact with the tumor. Bone infiltration is noted (white arrow). **g** Positron emission tomography/computed tomography scan with F-18 fluorodeoxyglucose showing standardized uptake values of 2.69

A 31-year-old woman presented with a posterior mediastinal mass on chest computed tomography (CT) scan during a medical check-up two years prior. She had left shoulder pain presentation to our hospital. Contrast-enhanced CT revealed a solitary and non-homogeneous 4-cm mass in the posterior mediastinum invading the left 2nd rib (Fig. 1a, b, c). Magnetic resonance imaging (MRI) revealed that signal inside the mass was equivalent to muscle tissue on the T1-weighted image, and was hyperintense on the T2-weighted image (Fig. 1d, e). Contrast-enhanced MRI showed similar signal changes and contrast effects on the second rib dorsal side in contact with the tumor. Bone infiltration was noted (Fig. 1f). Positron emission tomography/computed tomography scan with F-18 fluorodeoxyglucose suggested that the mass was associated with increased standardized uptake (max = 2.69) (Fig. 1g). All laboratory data were within normal ranges. CT-guided percutaneous fine-needle aspiration biopsy was performed, and she was diagnosed with a neurogenic tumor. We planned tumor resection by RATS combined with chest wall resection.

Under general anesthesia, the robot was positioned at the head of the operating table. The patient was intubated with a nerve integrity monitor electromyogram endotracheal tube for intraoperative neuromonitoring and place.

**Fig. 2:**
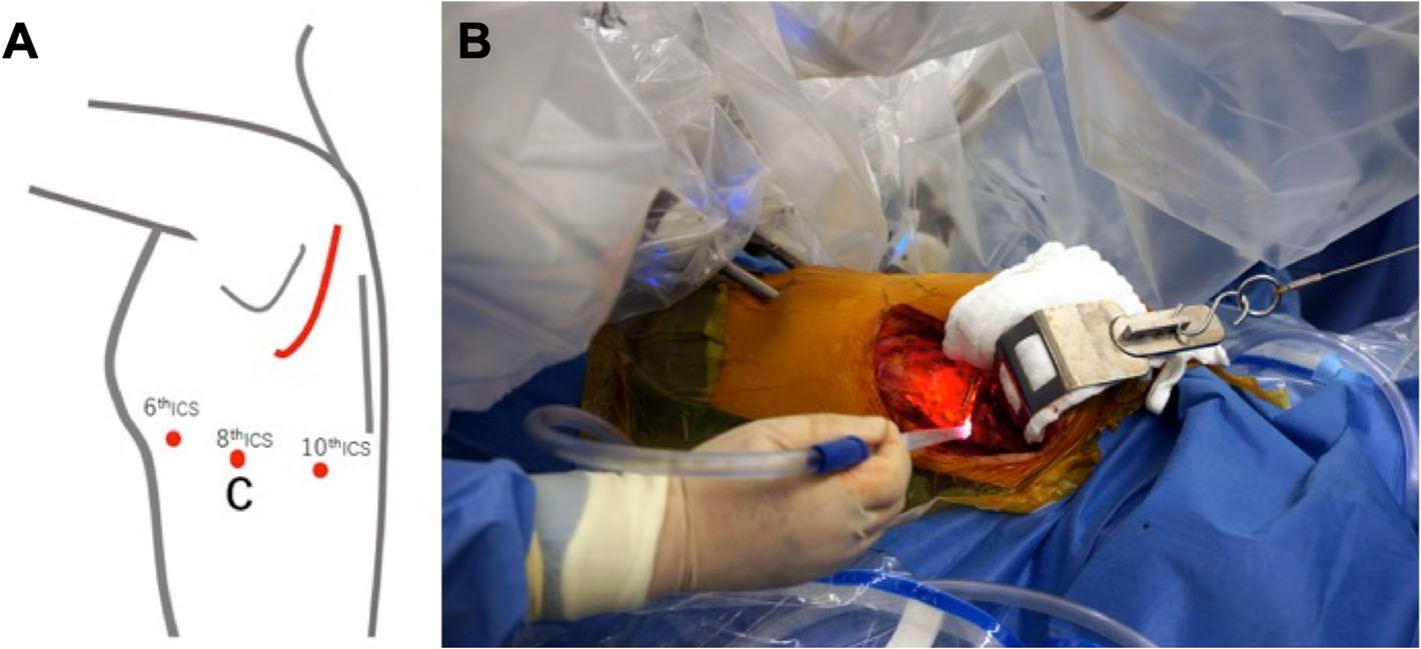
**a** Port placement (red circle) and posterior skin incision (red line). (ICS, Intercostal; C, Camera). **b** Intraoperative findings after the da Vinci surgical system was docked with the patient, and the left chest was explored

Robotic 8-mm ports were introduced (Fig. 2a). The da Vinci Xi® system (Intuitive Surgical, Sunnyvale, CA, USA) was then docked to the patient, and we explore the left pleural cavity (Fig. 2b). The tumor originated from the posterior mediastinum between the first and fourth rib, invading the posterior chest wall with no invasion of the vessels. The parietal pleura surrounding the tumor was resected with the robotic electrocautery, and both the second and third ribs were exposed proximally and distally. The edge was dissected from the lower edge of the first rib to the upper edge of the fourth rib. As the preoperative CT findings showed, the tumor invaded the second rib. The first and second intercostal muscles were dissected to secure the surgical margin. After that the second rib was cut down through the posterior incision, and the tumor was removed in its entirety, including the chest wall, from the thoracic cavity. After confirming hemostasis, the robot was undocked. A 21-F chest tube was inserted to the thoracic cavity through the camera port at the eighth intercostal space, and the wounds were closed. The postoperative course was uncomplicated. She has had no recurrences of the tumor after surgery.

**Fig. 3:**
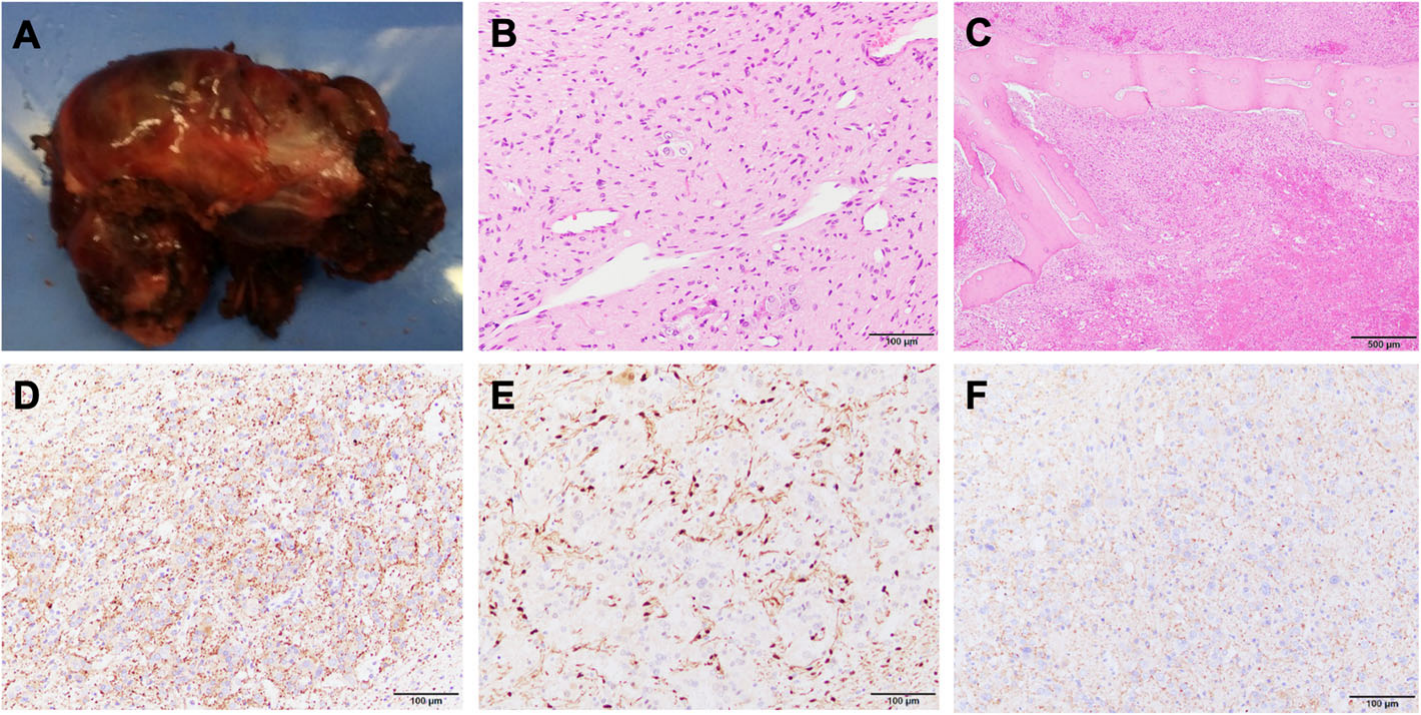
**a** Macroscopic findings of the resected specimen. The tumor consists of a tan red irregular ovoid mass measuring 4.5 × 3.3 × 2.2 cm. **b** The specimen is stained with hematoxylin-eosin; original magnification: × 20. The specimen is a nodular lesion continuous with the peripheral nerve, characterized by epithelial-like cells with abundant proliferating eosinophilic endoplasmic reticulum forming small clusters. There are a few atypical spindle cells in the background. **c** Rib bone tissue stained with HE revealing invasion; original magnification: × 4. The specimen is also stained with **d** S-100, **e** chromogranin and **f** synaptophysin; original magnification: × 20

Grossly, the resected tumor was a tan red-colored, irregular-surfaced and ovoid-shaped mass measuring 4.5 × 3.3 × 2.2 cm (Fig. 3a). Histologically, the tumor cells formed nests surrounded by a fine vascular network. The tumor cells ranged spindle to polygonal in shape. Their nuclei were spindle shape, and contained fine chromatin and inconspicuous nucleoli. The cytoplasm was abundant and eosinophilic. Marked atypism was absent (Fig. 3b). The tumor invaded the bony tissue of the rib included in the specimen (Fig. 3c). Immunohistochemically, the tumor cells were positive for chromogranin, and synaptophysin. Anti-S100 protein immunostain highlighted the sustentacular cells in the tumor, (Fig. 3d, e, f). Based on these findings, the tumor was diagnosed as paraganglioma with invasion to the rib. Because urinary adrenaline and metanephrine were within normal ranges, the tumor was considered to be nonfunctional. According to the grading system for adrenal pheochromocytoma and paraganglioma (GAPP) score [3], histological pattern was zellballen (0 point), cellularity was low (< 150 cells/HPF) (0 point), Ki-labeling index was 1–3% (1 point), and the tumor invaded the rib (2 point) and had non-functional (0 point). The total score was 3. An additional resection was not performed.

## Discussion and conclusions

Various approaches have been described for surgical treatment of neurogenic mediastinal tumors. These include thoracotomy [4, 5], neck approaches, transmanubrial approaches [6–8], thoracoscopic surgery [9, 10], and combinations of these procedures [11]. The da Vinci® robotic surgical system (Intuitive Surgical, Sunnyvale, CA, USA) enables highly accurate, minimally invasive procedures. In particular, the 3D imaging and multi-jointed forceps of the da Vinci® system allows procedures deep in the thoracic cavity to be performed much more easily than in conventional thoracoscopic surgery [1]. These advantages also enable reconstruction of vessels under thoracoscopic view. Suda et al. reported robot-assisted thymectomy with vascular replacement, they described that the robotic arms with movable joints similar to those of humans without physiological tremor facilitates vascular prosthetic replacement through end-to-end anastomosis [12].

In the present case, we performed tumor resection using RATS combined with chest wall resection. Although the mediastinum is tightly packed with intertwined organs and conduits, the wristed instruments of the robotic surgical system have increased range of motion and enabled the tumor resection without organ injury in the thoracic cavity. In addition, some authors reported the postoperative course of RATS. In comparison with conventional median sternotomy and VATS approach, RATS revealed lower amount of postoperative drainage, shorter duration of chest drainage and length postoperative hospital stay [13]. These results reflect less invasiveness of RATS.

Paragangliomas develop from chromaffin cells of the sympathetic nervous system distributing over the body [14]. Most mediastinal paragangliomas originate in the posterior portion [15]. Diagnosis of a functional paraganglioma requires urinary adrenaline or metanephrine levels three times higher than normal reference values. In this case, these values were within normal range. There is no established treatment other than surgical resection for paraganglioma [16, 17]. The prognosis is favorable in cases of complete resection, but the outcomes might be poor in malignant cases with residual tumor [18]. Even if tumor is histologically benign, it may clinically manifest a malignant behavior. Subsequently, long-term follow-up is mandatory for paraganglioma [19]. Although the tumor invaded the rib and the GAPP score in this case indicated histological grade was moderately differentiated type [3], successful resection was performed in our patient, and a favorable prognosis was expected.

In conclusion, RATS is a useful procedure, enabling safe and minimally invasive resection of mediastinal tumors invading the surrounding organs. Further applications in thoracic surgery are expected in the future.


**Additional file 1.** Intrathoracic view during the surgery. The parietal pleura surrounding the tumor was resected with the robotic electrocautery, the edge was dissected from the lower edge of the first rib to the upper edge of the fourth rib. The first and second intercostal muscles were dissected to secure the surgical margin. After that the second rib was cut down through the posterior incision, and the tumor was removed in its entirety, including the chest wall, from the thoracic cavity.


## Data Availability

Not applicable.

## Abbreviations

CT: Computed tomography
GAPP: Grading system for adrenal pheochromocytoma and paraganglioma
RATS: Robot-assisted thoracoscopic surgery
